# IL-6/STAT3 Axis Activates Glut5 to Regulate Fructose Metabolism and Tumorigenesis

**DOI:** 10.7150/ijbs.68990

**Published:** 2022-05-16

**Authors:** Xiaoke Huang, Jing Fang, Weiqi Lai, Yu Hu, Liang Li, Yuanyou Zhong, Shiwei Yang, Dan He, Rui Liu, Qingfeng Tang

**Affiliations:** 1 Department of Urology, Xindu district People's hospital of Chengdu, Chengdu, 610500, China; 2 Department of Nephrology, The sixth people's hospital of Chengdu, Chengdu, 610051, China; 3 The Second Affiliated Hospital of Chengdu Medical College, China National Nuclear Corporation 416 Hospital, Chengdu, Sichuan, P. R. China; 4 State Key Laboratory of Oral Diseases, National Clinical Research Center for Oral Diseases, Research Unit of Oral Carcinogenesis and Management, Chinese Academy of Medical Sciences, West China Hospital of Stomatology, Sichuan University, Chengdu, Sichuan 610041, P. R. China

**Keywords:** IL-6, STAT3, Glut5, fructose metabolism, tumorigenesis

## Abstract

Cancer cells frequently use fructose as an alternative energy and carbon source, to fuel glycolysis and support the synthesis of various biomacromolecules. Glut5 is the only fructose-specific transporter, which lacks the ability to transport other carbohydrates such as glucose and galactose. Interplay between inflammatory factors and cancer cells renders inflammatory tissue environment as a predisposing condition for cancer development. Nevertheless, how inflammatory factors coordinate with fructose metabolism to facilitate tumor growth remains largely elusive. Here we show that treatment with IL-6 activates fructose uptake and fructolysis in oral squamous cell carcinoma (OSCC) cells and prostate cancer cells. Mechanistic study shows that transcription factor STAT3 associates with Glut5 promoter region and enhances Glut5 transcription in response to IL-6 treatment. Knockdown of Glut5 abolished IL-6-induced fructose uptake and utilization of fructose, and compromises IL-6-elicited tumor cell proliferation. Further, positive correlation between Glut5 and IL-6 expression is observed in multiple cancers. Our findings demonstrate a regulatory cascade underlying the crosstalk between inflammation and fructose metabolism in cancer cells, and highlights Glut5 as a novel oncogenic factor.

## Introduction

**Figure 1:**
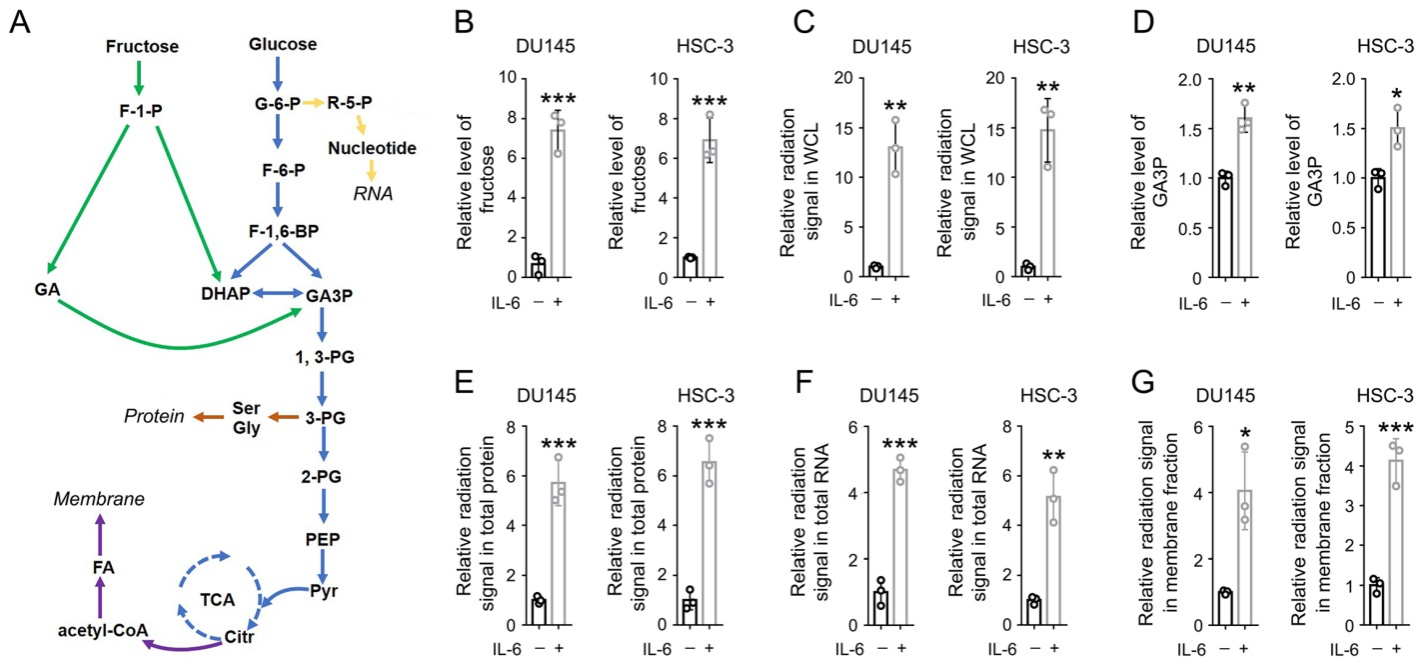
** IL-6 activates both the uptake and utilization of fructose.** (A) A schematic of fructose metabolism. Distinct metabolic pathways are shown with arrows in different colors: glycolysis and TCA cycle in blue, fructolysis pathway in green, nucleotide and RNA synthesis in yellow, fatty acid and membrane biosynthesis in purple, and amino acids and protein biosynthesis in brown. (B, D) DU145 and HSC-3 cells were stimulated with IL-6 (50 ng/mL) for 24 h, and the level of cellular fructose (B) and GA3P (D) was measured. Data represent the mean and SD from three independent experiments. *P < 0.05; **P < 0.01; ***P < 0.001. (C, E-G) DU145 and HSC-3 cells were stimulated with IL-6 (50 ng/mL) for 24 h, and then incubated with 10 µCi [^14^C]-fructose for 30 min (C) or 1 h (E-G). The level of radiation signal in whole cell lysate (B), total protein (E), total RNA (F) or cell membrane fraction (G) was measured. Data represent the mean and SD from three independent experiments. *P < 0.05; **P < 0.01; ***P < 0.001.

Tumor cells take advantage of the metabolic adaptation of fructose for their malignant growth, particularly to compensate for the glucose deficiency in the tumor microenvironment 1. Like glucose, fructose can be metabolized to support the synthesis of nucleotides, amino acids and fatty acids. After entering cells, fructose is rapidly phosphorylated by ketohexokinase (also known as fructokinase) to generate fructose 1-phosphate (F1P). F1P is then cleaved into dihydroxyacetone phosphate (DHAP) and glyceraldehyde by aldolase B. The latter is phosphorylated by propyl kinase to produce glyceraldehyde 3-phosphate (GA3P), which eventually joins glycolysis (Figure 1A) 2.

Glut transporters are the members of the solute transporter 2 (SLC2) family, with a total of 14 different proteins identified so far, which can promote the diffusion of glucose or fructose across cell membranes in a concentration-dependent manner 3. Compared to other family members, Glut5 is considered specific for fructose, with a negligible ability to transport glucose or galactose. High Glut5 expression is found in the luminal membrane of intestinal cells, and its modest expression is also observed in brain, adipose tissue, kidney, testis and skeletal muscle 2, 3. However, the expression pattern of Glut5 during tumor development remains elusive.

The discovery of the interconnection between inflammation and cancer development could be traced back to 1863, when Rudolf Virchow observed that chronic inflammatory sites were frequently the origin of malignant tumors 4. So far, a large body of studies demonstrates that inflammation does play a huge role in cancer development, whereas it can also be used as a targeted strategy to cure cancer 5. Inflammatory conditions can activate or accelerate carcinogenic transformation, and malignant cells with genetic and epigenetic alterations, in turn, can generate an inflammatory microenvironment that further contributes to tumor progression 6. Interleukin 6 (IL-6)/Signal Transducer and Activator of Transcription 3 (STAT3) signaling axis has been considered as an essential intrinsic pathway of cancer inflammation 7. IL-6 stimulation leads to Janus Kinase 2 (JAK2)-dependent phosphorylation of cytoplasmic STAT3, which facilitates STAT3 dimerization and translocation to the nucleus to function as a transcriptional factor 8, 9. In cancer cells, abnormal IL-6 expression and constitutive hyperactivation of STAT3 are closely correlated with an enhanced cancer cell proliferation and drug resistance. Nevertheless, the crosstalk between IL-6/STAT3 axis and fructose metabolism is still relatively unknown.

Here, we demonstrate that, in response to IL-6 treatment, STAT3 enhances the transcription of Glut5 by associating with its promoter region. IL-6/STAT3 axis-mediated Glut5 expression enhances fructose uptake and utilization, and promotes tumor cell growth.

## Materials and Methods

Antibodies recognizing Glut5 (ab279363), Ki67 (ab16667), and recombinant human IL-6 protein (ab9627) were obtained from Abcam. Antibodies recognizing STAT3 (#9139), and STAT3-pY705 (#9145) were obtained from Cell Signaling Technology. ^14^C_6_-fructose was purchased from Biotrend, Köln (#MC1459-50).

### DNA constructs and mutagenesis

Human IL-6, and STAT3 were amplified and inserted into pcDNA3.1 or SFB-pCDH vector. Mutant Glut5 promoter constructs were prepared as follows: STAT3-mut, 'TTCCAGGAA' into 'AAAAAGGAA'; SOX2-mut, 'AAACAAA' into 'GCGAGCG'; PPARγ-mut, 'TGTTCTTTCACC' into 'TGCGCCGCAGTC'.

shRNAs were prepared using the following sequences: STAT3 shRNA, TAC CTA AGG CCA TGA ACT T (targeting non-coding region); Glut5 shRNA-1, TTG GCT CTA AAC AAA TGC C; Glut5 shRNA-2, TAT GTT GTT GAA CAG CAA G.

### Cell culture

HSC-3 cell was purchased from JCRB Cell Bank. DU145 cell was purchased from ATCC. Both the two cell lines were maintained with Dulbecco's Modified Eagle Medium (Cat No: 11885076, ThermoFisher Scientific).

### Semi-quantitative reverse PCR

Primers used for semi-quantitative reverse PCR were synthesized using the following sequences: Glut5-F: GGCATCCTTGTGGCCCAGATC; Glut5-R: CTGTGTCCTGCAGTGCCAGAG; Actin-F: CATGTACGTTGCTATCCAGGC; Actin-R: CTCCTTAATGTCACGCACGAT.

### ChIP-PCR assay

ChIP-PCR assay was performed using an Upstate Biotechnology kit, as described previously 10. The specific primers used in PCR were synthesized using the following sequences: 5-CTAGCTTGGTGCTCTGTCCTGTG-3 (forward) and 5-CTGTAGTAATAGAATTGGGAACAAGACCTG C-3 (reverse).

### Colony formation assay

Colony formation assay was performed following previous report 11. Briefly, 50 cells were seeded in a six-well plate, followed by continuous culture for 14 days. The clones were then fixed and visualized by Crystal Violet solution. Clones with more than 50 cells were counted.

### Measurements of metabolites

Cellular level of GA3P is measured by using Glyceraldehyde 3-Phosphate Assay Kit (Fluorometric) obtained from Abcam (ab273344). Cellular level of fructose is measured by using Fructose Assay Kit obtained from Abcam (ab83380).

### Measurements of ^14^C-fructose metabolism

Cell plasma membrane was isolated by using OrgFrontier™ Plasma Membrane Isolation Kit obtained from BioVision (K414). Total RNA was isolated using PureLink™ RNA mini kit obtained from ThermoFisher Scientific (12183018A). Total protein samples were prepared from total cell lysates via precipitation by trichloroacetic acid (TCA)/acetone. The radiation signal was measured using scintillation counting, and normalized to cell number.

### Mouse tumor xenograft model

5 × 10^6^ tumor cells were injected into the back of 6-week-old male BALB/c nude mice. At the indicated time points, the tumor size was measured (volume = 1/2 × length × width^2^), as previously reported 12. The use of animals was approved by the institutional review board of West China Hospital of Stomatology (OSCC mouse model) and Second Affiliated Hospital of Chengdu Medical College, China National Nuclear Corporation 416 Hospital (prostate cancer mouse model).

### Clinical tumor samples and evaluation of immunohistochemical staining

Clinical samples were obtained with informed consent from all subjects. The use of human OSCC specimens was approved by the Institutional Review Board at West China Hospital of Stomatology. The use of human colorectal cancer and glioma specimens was approved by the Institutional Review Board at the Second Affiliated Hospital of Chengdu Medical College, China National Nuclear Corporation 416 Hospital. The use of human prostate cancer specimens was approved by the Institutional Review Board at the Xindu district People's hospital of Chengdu.

To obtain the immunohistochemical staining score, a proportion score and an intensity score were multiplied, as previously reported 13. The proportion scores were calculated as: 0, none positive staining; 1, 0-10%; 2, 11-30%; 3, 31% to 70%; 4, 71-100%. The intensity scores were calculated as: 0, negative; 1, weak; 2, moderate; 3, strong; 4, very strong.

### Quantification and Statistical Analysis

Statistical analyses were performed using two-sided Student's t test or ANOVA test. All data represent the mean and SD of at least three independent experiments. Differences in means were considered statistically significant at *P* < 0.05. Bonferroni correction was used for the multiple hypothesis correction.

## Results

### IL-6 activates the uptake and utilization of fructose

To determine the impact of inflammation on fructose metabolism in cancer cells, prostate cancer DU145 cells and oral squamous cell carcinoma (OSCC) HSC-3 cells were cultured with DMEM medium containing 10 mM fructose. The cells were treated with active recombinant IL-6 protein for 24 h. We found that the level of intracellular fructose was markedly elevated after IL-6 treatment in either DU145 or HSC-3 cells (Figure 1B). Consistently, IL-6 treatment elevated the radiation signal in the whole cell lysates by more than 10 folds, after incubation with [^14^C]-fructose for 30 min (Figure 1C). Fructose could be converted into GA3P during fructolysis, and further used for the biosynthesis of protein, RNA, and phospholipids in membranes 2. As expected, a roughly 1.5-fold increase in GA3P level was also detected (Figure 1D), hinting that IL-6 treatment enhanced fructolysis. Further, a markedly stronger radiation signal was detected in total protein, total RNA, and cell membrane samples derived from IL-6-treated cells (Figure 1E-1G). These data suggest that IL-6 treatment strengthens both the uptake and utilization of fructose.

### STAT3 associates with Glut5 promoter and activates Glut5 transcription in response to IL-6

**Figure 2:**
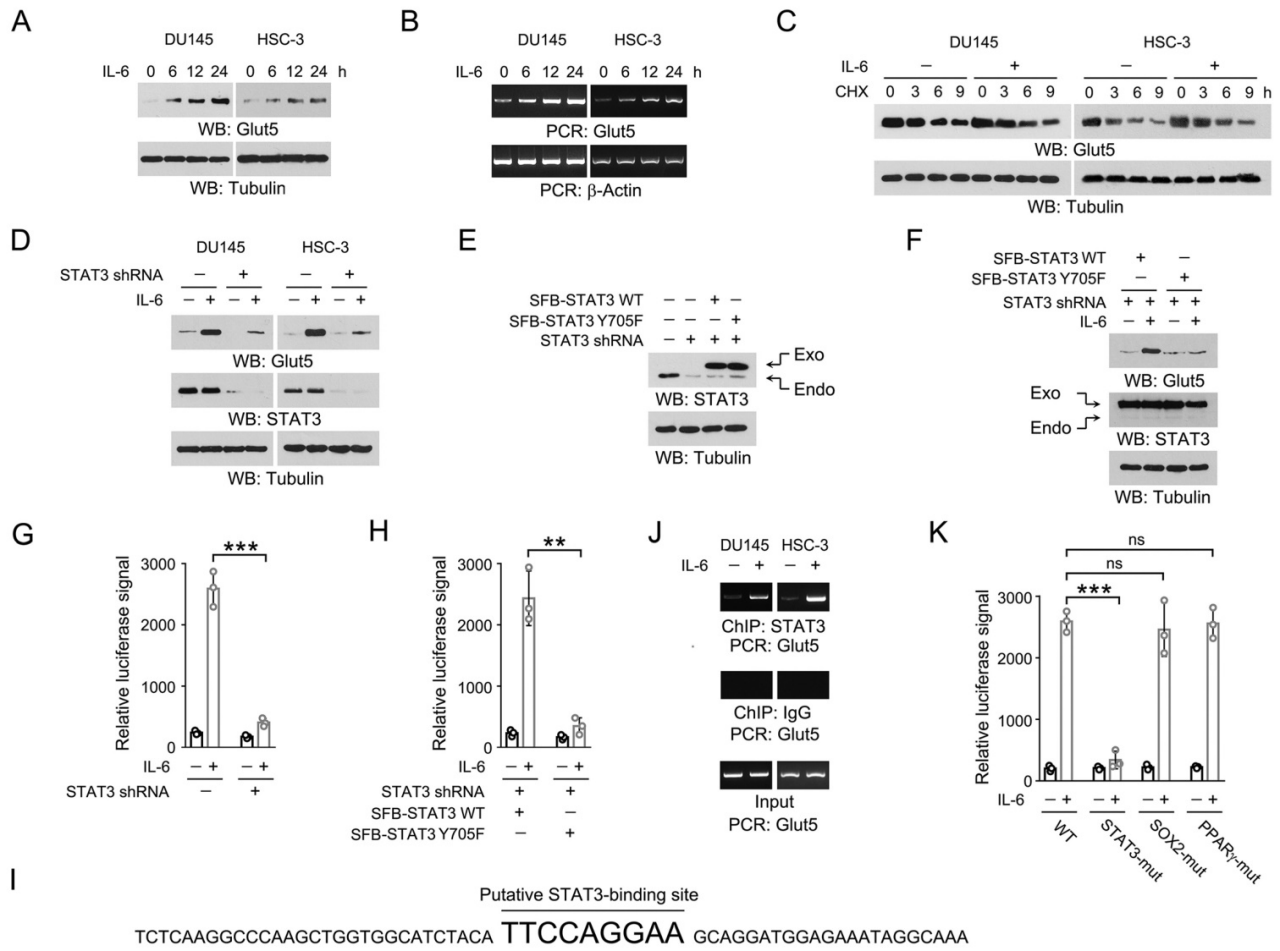
** STAT3 associates with Glut5 promoter and activates Glut5 transcription in response to IL-6.** (A-B) DU145 and HSC-3 cells were stimulated with IL-6 (50 ng/mL) for the indicated periods of time. Expression of Glut5 was examined by immunoblot (A) and RT-PCR (B). (C) DU145 and HSC-3 cells were treated with CHX (100 μg/ml) for the indicated periods of time in presence or absence of 50 ng/mL IL-6, and expression of Glut5 was examined by immunoblot. (D) DU145 and HSC-3 cells expressed with STAT3 shRNA were stimulated with IL-6 (50 ng/ml) for 24 h. Immunoblot using indicated antibodies was performed. (E-F) HSC-3 cells were expressed with STAT3 shRNA, SFB-STAT3 WT or SFB-STAT3 Y705F (E). These cells were then stimulated with IL-6 (50 ng/mL) for 24 h (F). Immunoblot using indicated antibodies was performed. STAT3 shRNA targets the non-coding region. (G) HSC-3 cells expressed with STAT3 shRNA and luciferase reporter construct were stimulated with IL-6 (50 ng/mL) for 9 h. Luciferase assay was performed. Data represent the mean and SD from three independent experiments. ***P < 0.001. (H) HSC-3 cells with expression of STAT3 shRNA, SFB-STAT3 WT or SFB-STAT3 Y705F were expressed with luciferase reporter construct, and then stimulated with IL-6 (50 ng/mL) for 9 h. Luciferase assay was performed. Data represent the mean and SD from three independent experiments. **P < 0.01. (I) The putative STAT3-binding site and its franking sequences in Glut5 promoter region is shown. (J) DU145 and HSC-3 cells were stimulated with IL-6 (50 ng/mL) for 2 h. ChIP-PCR was performed. (K) HSC-3 cells expressed with WT and the indicated mutant luciferase reporter constructs were stimulated with IL-6 (50 ng/mL) for 9 h. Luciferase assay was performed. Data represent the mean and SD from three independent experiments. ***P < 0.001; ns, not significant.

Glut5 transporter is specific for fructose 14. Of our particular interest, we examined the expression of Glut5 upon IL-6 stimulation. Time course analyses revealed an initial increase in Glut5 expression after 6 h incubation with IL-6, which was further extended at 12 and 24 h (Figure 2A-2B). The enhanced Glut5 expression was dynamically consistent at both protein and mRNA levels, suggesting that IL-6 induced Glut5 expression by increasing its gene transcription. This observation was further supported by the protein stability assay using chlorhexidine, revealing that the protein degradation rate of Glut5 was barely changed after IL-6 treatment (Figure 2C).

We then wondered at the key factors involved in IL-6-induced Glut5 expression. IL-6 triggers JAK2-dependent phosphorylation of STAT3 Y705, which facilitates STAT3 dimerization and translocation into nucleus to initiate the transcription of a wide spectrum of genes 8. Indeed, knockdown of STAT3 largely abolished Glut5 expression in the IL-6-treated DU145 and HSC-3 cells (Figure 2D). In line with this, reconstituted expression of the non-phosphorylatable STAT3 Y705F mutant in the endogenous STAT3-depeleted HSC-3 cells also compromised IL-6-induced Glut5 expression (Figure 2E-2F). To further examine the impact of STAT3 on the transcription efficiency of Glut5, we prepared a reporter construct where the expression of luciferase was under the control of the promoter sequence of Glut5 (-2000 ~ 0). As expected, IL-6 treatment markedly enhanced the luciferase signal, which could be substantially eradicated by knockdown of STAT3 or reconstituted expression of STAT3 Y705F mutant (Figure 2G-2H). Analysis of the sequence of Glut5 promoter region revealed a putative STAT3-binding site 'TTCCAGGAA', which is similar to the reported STAT3-responsive sequence (Figure 2I) 15. This assumption was confirmed by ChIP-PCR assay using the primers targeting the franking sequences of this site (Figure 2J). Further, disruption of this STAT3-responsive motif by replacing 'TTCCAGGAA' into 'AAAAAGGAA' (STAT3-mut) largely blocked IL-6-evoked luciferase expression; in contrast, mutation of the putative SOX2 or PPARγ-binding site caused only minor effects (Figure 2K). These results suggest that STAT3 binds to Glut5 promoter and activates Glut5 transcription upon IL-6 treatment.

### IL-6-induced Glut5 expression promotes fructose utilization

**Figure 3:**
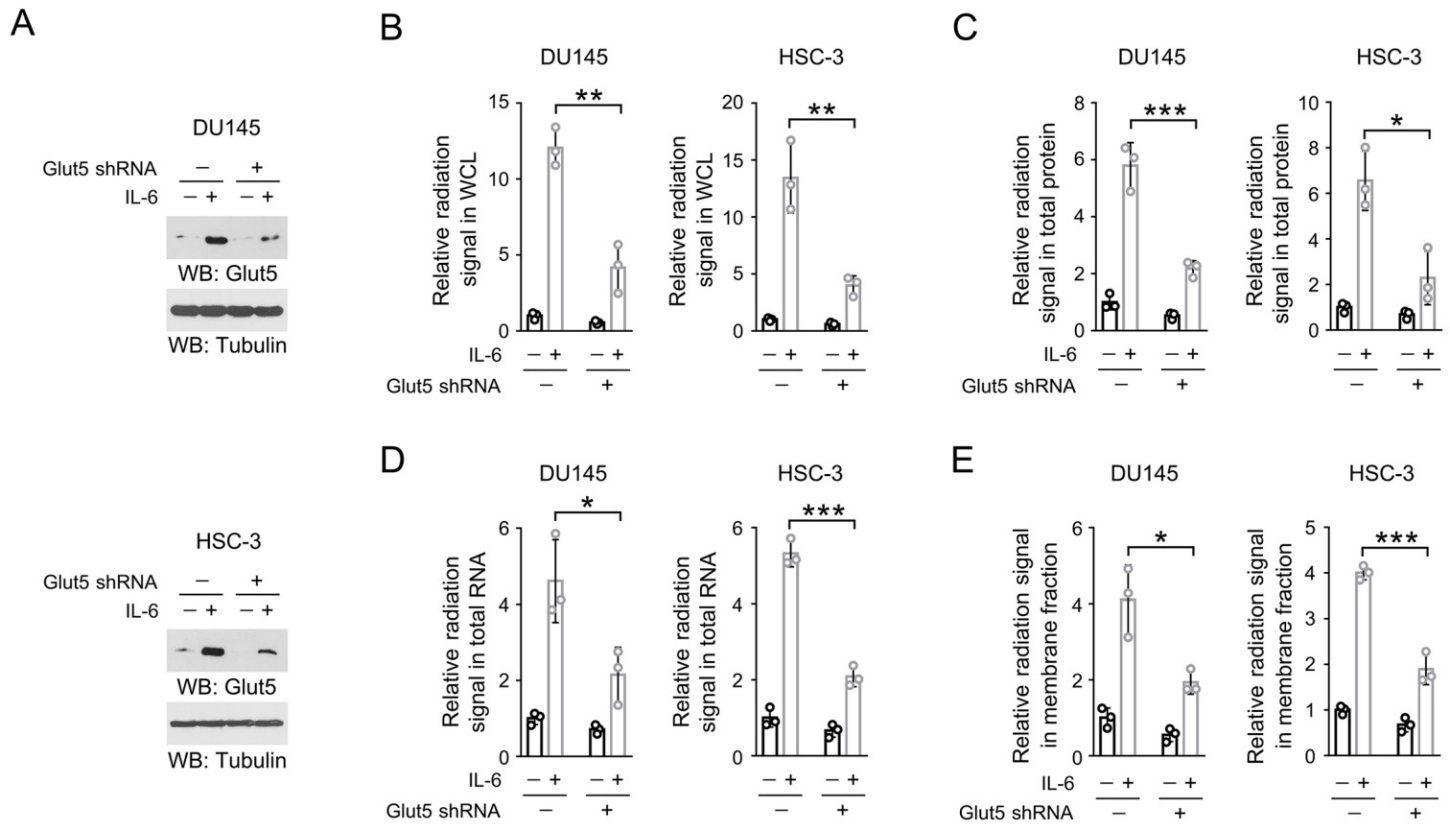
** IL-6-induced Glut5 expression promotes fructose utilization.** (A) DU145 (upper panel) and HSC-3 (bottom panel) cells expressed with Glut5 shRNA were incubated with 50 ng/mL IL-6 for 24 h. Immunoblots were performed with indicated antibodies. (B-E) DU145 and HSC-3 cells with expression of Glut5 shRNA were stimulated with IL-6 (50 ng/mL) for 24 h, and then incubated with 10 µCi [^14^C]-fructose for 30 min (B) or 1 h (C-E). The level of radiation signal in whole cell lysate (B), total protein (C), total RNA (D) or membrane fraction (E) was measured. Data represent the mean and SD from three independent experiments. **P < 0.01; ***P < 0.001.

Next, we turned to examine the effect of IL-6-mediated Glut5 expression on fructose metabolism. We knockdown the endogenous expression of Glut5 in IL-6-treated DU145 and HSC-3 cells (Figure 3A), and incubated these cells with ^14^C-labeled fructose. Notably, loss of Glut5 abolished roughly 80% IL-6-induced radiation signal in the whole cell lysates (Figure 3B), hinting that Glut5 was likely the major fructose transporter in the IL-6-stimulated DU145 and HSC-3 cells. Similarly, knockdown of Glut5 markedly eliminated the levels of ^14^C-labeled cellular protein, RNA or membrane fraction (Figure 3C-3E). These results suggest that IL-6-induced Glut5 expression promotes fructose utilization.

### IL-6-induced Glut5 expression promotes OSCC and prostate cancer cell growth

**Figure 4:**
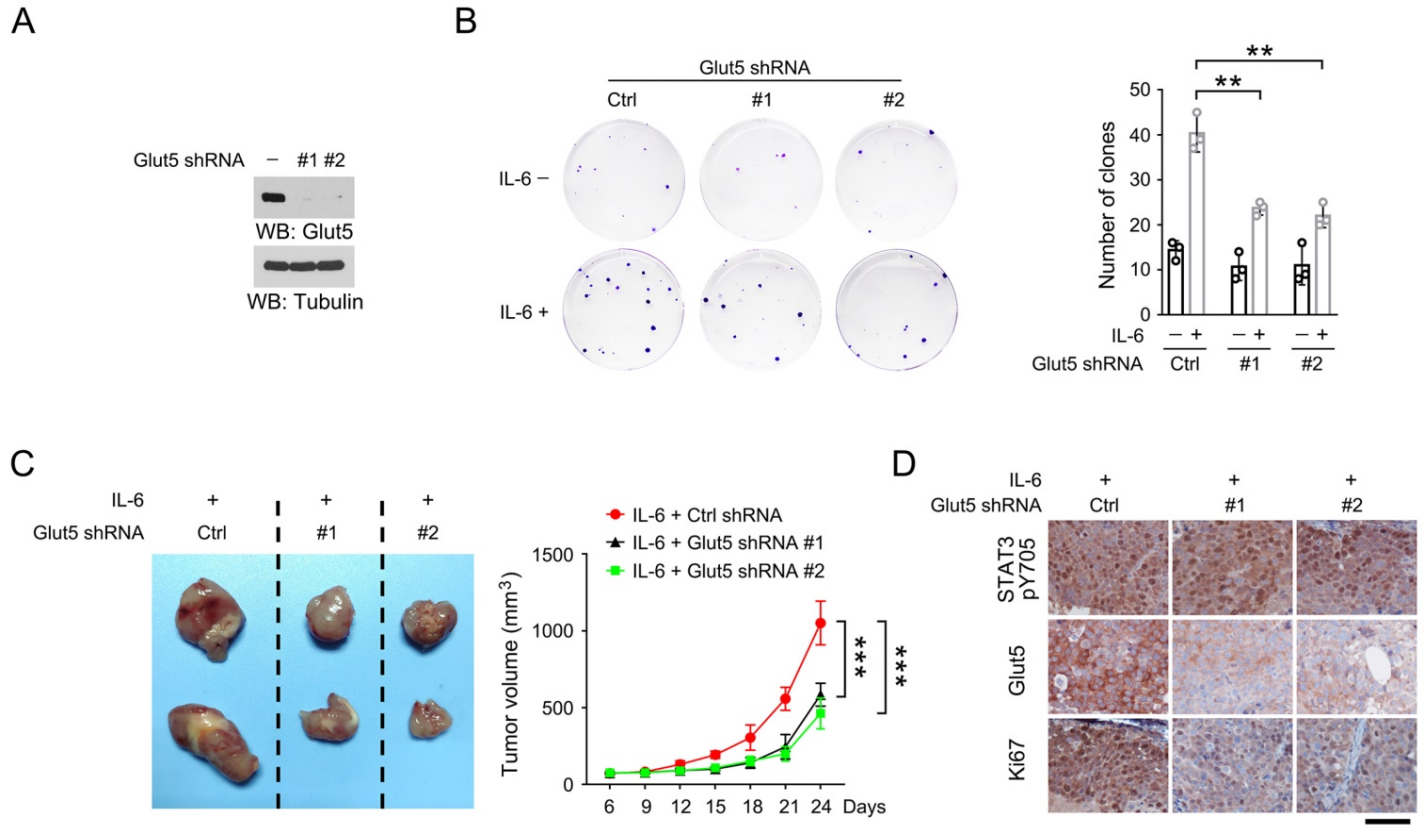
** IL-6-induced Glut5 expression promotes tumorigenesis.** (A) HSC-3 cells were stably expressed with distinct Glut5 shRNAs. Immunoblots were performed with indicated antibodies. (B) 50 HSC-3 cells stably expressed with control shRNA or Glut5 shRNA were seeded, and continuously cultured for 14 days in presence or absence of 50 ng/mL IL-6. The clone numbers were counted. **P < 0.01. (C) HSC-3 cells with stable expression of IL-6 or Glut5 shRNA were subcutaneously injected in mice. The images of two representative xenografts from each group were shown (left panel). The volume of mice tumor xenografts (n = 7) was measured at indicated time points after injection (right panel). Data represent the mean and SD. ***P < 0.001. (D) Immunohistochemical staining of mouse xenografts samples was performed with indicated antibodies. Scale bar, 50 µm.

To determine the impact of IL-6-induced Glut5 expression on tumor cell proliferation, we established HSC-3 cells with stable Glut5 knockdown using two distinct shRNAs (Figure 4A). Colony formation assay revealed that treatment with IL-6 induced apparently more cell clones after 14-day continuous culture, while Glut5 knockdown substantially decreased the IL-6-induced colony formation (Figure 4B). Interestingly, loss of Glut5 caused only minor effects on the colony formation in the absence of IL-6, which may suggest that fructose uptake by Glut5 at its basal expression level could be compensated by other transports.

We then established the mouse xenograft model by subcutaneously injecting HSC-3 cells that were stably expressed with IL-6 and Glut5 shRNAs. As expected, expression of Glut5 shRNAs palpably retarded tumor growth (Figure 4C). Similar results were also observed in mouse xenografts formed by DU145 cells with or without expression of Glut5 shRNA (Figure S1A-S1B). By immunohistochemical staining, we found a substantially decreased expression of proliferative cell marker Ki67 in Glut5 shRNA-expressed tumor cells compared to the control shRNA-expressed counterparts; in contrast, phosphorylation of STAT3 Y705, a marker for activated STAT3, could be equally detected in all groups (Figure 4D and S1C). IL-6-induced Glut5 expression promotes OSCC and prostate cancer cell growth.

### Glut5 expression correlates with clinical aggressiveness of multiple human cancers

**Figure 5:**
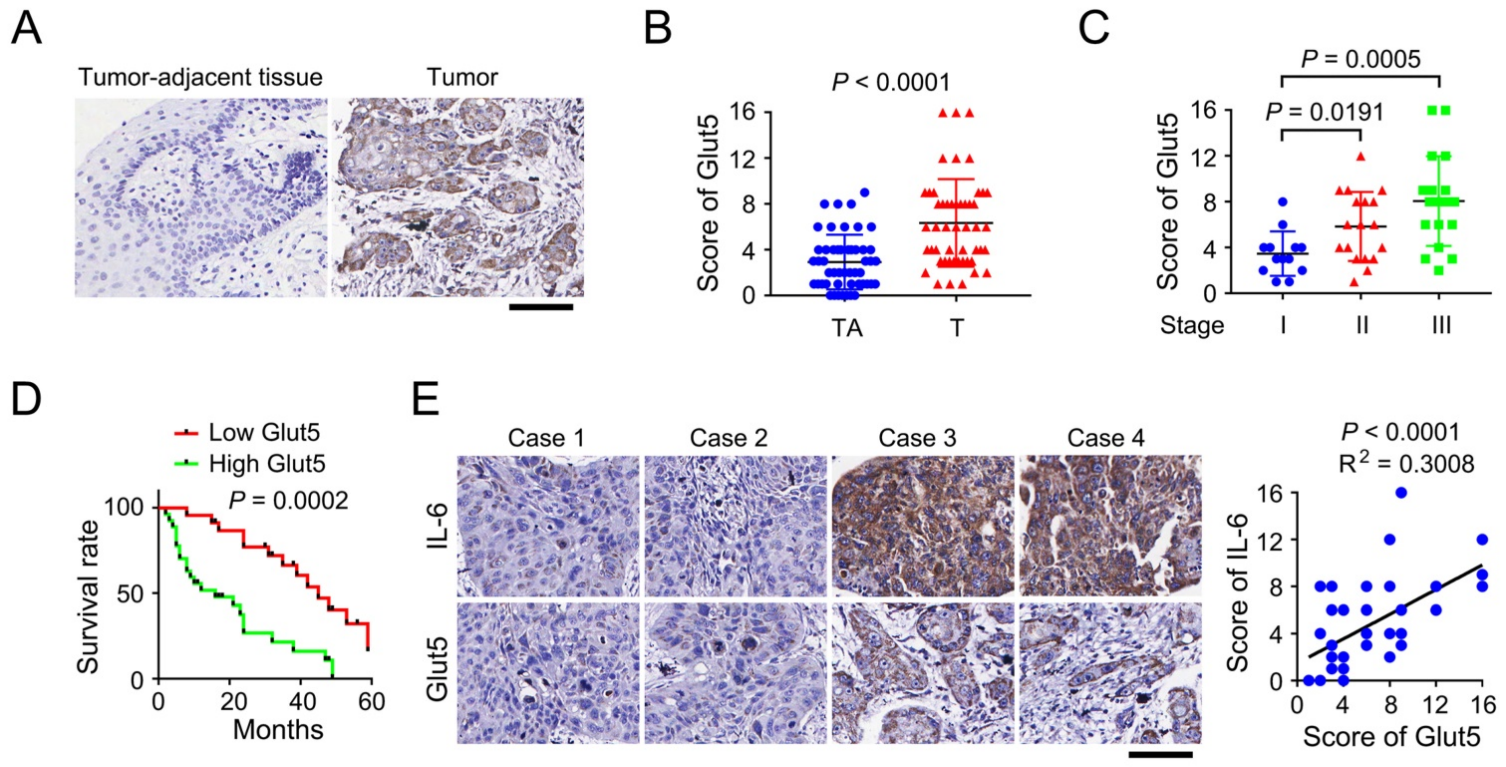
**Glut5 expression correlates with clinical aggressiveness of OSCC.** (A) Immunohistochemical staining was performed using tumor-adjacent tissues (n = 50) and human OSCC tumor tissues (n = 50) with an anti-Glut5 antibody. Scale bar, 80 µm. (B-C) Immunohistochemical staining scores were compared between tumor-adjacent tissues and tumor tissues (B), or among the tumors diagnosed at different stage (C). TA, tumor-adjacent tissue; T, tumor tissue. (D) Kaplan-Meier plots of the overall survival time of OSCC patients with high or low Glut5 expression was performed. *P* value was calculated using the log-rank test. (E) Immunohistochemical staining were performed using human OSCC samples with indicated antibodies (left panel), and the immunohistochemical staining scores were analyzed by linear regression (right panel). Scale bar, 80 µm.

To evaluate the clinical relevance of Glut5 expression, we performed immunohistochemical staining using clinical samples that were surgically derived from OSCC patients. We found that expression of Glut5 was up-regulated in OSCC tumor tissues versus the non-tumorous counterpart tissues (Figure 5A-5B). In addition, OSCC tumors diagnosed as Stage II or III showed a higher Glut5 expression than those tumors at Stage I, though no significant difference was noticed between Stage II and III tumors (Figure 5C). Analyses of the follow-up records of 50 OSCC patients revealed a markedly longer average survival time for the patients with low Glut5 expression (Figure 5D). Further, those tumor cases harboring high level of IL-6 expression were frequently companied with high Glut5 expression (Figure 5E). This correlation was further shown to be statistically significant by linear regression analyses (Figure 5E).

To expand our findings, we also examined the clinical relevance of Glut5 expression in prostate cancer, colorectal cancer and glioma. A positively corelated expression pattern of IL-6 and Glut5 was found in all the three tumor types (Figure S2A-S2C). Further, consistent with the observation in OSCC, the prostate cancer patients with low Glut5 expression showed a markedly longer survival time (Figure S2D). Due to the lack of the follow-up information of colorectal cancer and glioma patients, we analyzed the TCGA datasets via Human Protein Atlas website (https://www.proteinatlas.org/), and found that Glut5 was highly expressed in the colorectal cancer patients with poor outcome (Figure S2E), while the result was not statistically significant for glioma patients (Figure S2F). These results suggest that Glut5 expression correlates with clinical aggressiveness of multiple human cancers.

## Discussion

Tumor cell metabolism is programmed to consume large amounts of glucose, frequently resulting in glucose depletion in the tumor microenvironment 16. To compensate for the glucose deficiency, cancer cells utilize fructose as an alternative energy and carbon source, to fuel glycolysis and support the synthesis of various biomacromolecules. Accumulating evidences have shed a light on the aberrant fructose metabolism in human cancers. It is documented that increased fructose influx in pancreatic cancer cells reinforces nucleotide synthesis via the non-oxidizing pentose-phosphate pathway 17. Excess fructose consumption promoted colon cancer metastasis to liver 18. Among the hexose transporters, Glut5 shows a high affinity to fructose over glucose or galactose. Strikingly, high Glut5 expression was found in glioma cells, acute myeloid diseases and breast cancer cells, and aberrant Glut5 expression was associated with malignant progression and poor patient survival, revealing the potential involvement of Glut5 in tumorigenesis 19-21. In current work, we demonstrate that knockdown of Glut5 markedly blocked tumor cell proliferation in IL-6-expressed OSCC and prostate cancer xenografts. Further, the expression of Glut5 and IL-6 was found to be positively corelated in clinical samples of OSCC, prostate cancer, colorectal cancer and glioma, suggesting that the IL-6-medited Glut5 expression probably widely exist in multiple human cancers. Of note, high Glut5 expression was found to be associated with a poor patient outcome for OSCC, prostate cancer and colorectal cancer patients; while the difference of the survival duration is not statistically significant for glioma patients. Therefore, more work is still needed to determine the role of Glut5 in carcinogenesis.

Inflammation is an ancient and conserved biological process during evolution, which plays an essential role in tissue maintenance, repair and regeneration. In contrast, chronic inflammation is an uncontrolled form of inflammation that are evidenced to be involved in the development of many complex diseases and disorders 22. Accumulating evidences have pointed that chronic inflammation increases cancer risk, affecting all steps during carcinogenesis, such as inducing genomic instability and epigenetic modification, enhancing angiogenesis, and suppressing immune surveillance by modulating immune cells 23. In current data, we report an important and IL-6/STAT3 axis-dependent molecular mechanism that modulates Glut5 expression. We demonstrate that IL-6 treatment enhanced fructose uptake and promoted fructolytic flux toward the synthesis of amino acids, fatty acids and nucleotides. Inhibition of STAT3, by either knockdown or Y705F mutation, eradicated IL-6-induced Glut5 expression and Glut5 promoter-guided luciferase expression. Further, we identified a 'TTCCAGGAA' sequence within the promoter region of Glut5, which is similar to the previously reported STAT3-binding sequence 15. We also show that IL-6 treatment induced the recruitment of STAT3 to this site, and mutation of this site disrupted IL-6-induced luciferase activity. The current data adds to the knowledge of the regulatory role of inflammation factors on fructose metabolism, and may lead to a better understanding of the action model of some FDA-approved compounds with STAT3-inhibitory property, such as Pyrimethamine and Celecoxib 24. Considering several IL-6 inhibitors are currently available in clinical use for treating inflammatory and autoimmune diseases, such as tocilizumab, sarilumab, and satralizumab, the presented IL-6-meidated mechanism in our data might suggest the potential translational values of these IL-6 inhibitors in cancer treatment.

Excess consumption of fructose is associated with a risk for multiple diseases, including cancer. In the present work, our data reports a new regulatory mechanism involved in inflammation-directed metabolic reprogramming in cancer cells. STAT3 boosts Glut5 transcription by binding to its promoter region upon IL-6 stimuli. Activation of this IL-6/STAT3 axis-guided fructose metabolism promotes tumor cell growth.

## Supplementary Material

Supplementary figures.

## Abbreviations

OSCC: oral squamous cell carcinoma
GA3P: glyceraldehyde 3-phosphate
IL-6: Interleukin 6
STAT3: Signal Transducer and Activator of Transcription 3
F1P: fructose 1-phosphate
