# Supplementary material for: IL-6/STAT3 Axis Activates Glut5 to Regulate Fructose Metabolism and Tumorigenesis
Source: Int J Biol Sci. 2022 May 16;18(9):3668–75. doi: 10.7150/ijbs.68990 (PMC9254469; doi:10.7150/ijbs.68990)
Supplement: Supplementary file 1 — Supplementary figures. [file ijbsv18p3668s1.pdf]

Supplementary figure legends

Figure S1. Knockdown of Glut5 expression impairs tumorigenesis

(A) DU145 cells were stably expressed with distinct Glut5 shRNAs. Immunoblots were performed with indicated antibodies.

(B) DU145 cells with stable expression of IL-6 and Glut5 shRNA were subcutaneously injected in mice. The images of two representative xenografts from each group were shown (left panel). The volume of mice tumor xenografts (n = 7) was measured at indicated time points after injection (right panel). Data represent the mean and SD. ***$P < 0.001$.

(C) Scores of the immunohistochemical staining in Figure 4D.

Figure S2. Glut5 expression correlates with clinical aggressiveness of multiple cancers

(A-C) Immunohistochemical staining were performed using human prostate cancer (A), colorectal cancer (B), and glioma (C) samples with indicated antibodies (left panel), and the immunohistochemical staining scores were analyzed by linear regression (right panel). Scale bar, 80 μm (A); Scale bar, 80 μm (B); Scale bar, 150 μm (C).

(D-F) Kaplan-Meier plots of the overall survival time of human prostate cancer (D), colorectal cancer (E), and glioma (F) samples with high or low Glut5 expression was performed. *P* value was calculated using the log-rank test. The results of (E) and (F) were generated by Human Protein Atlas website.

Figure S1

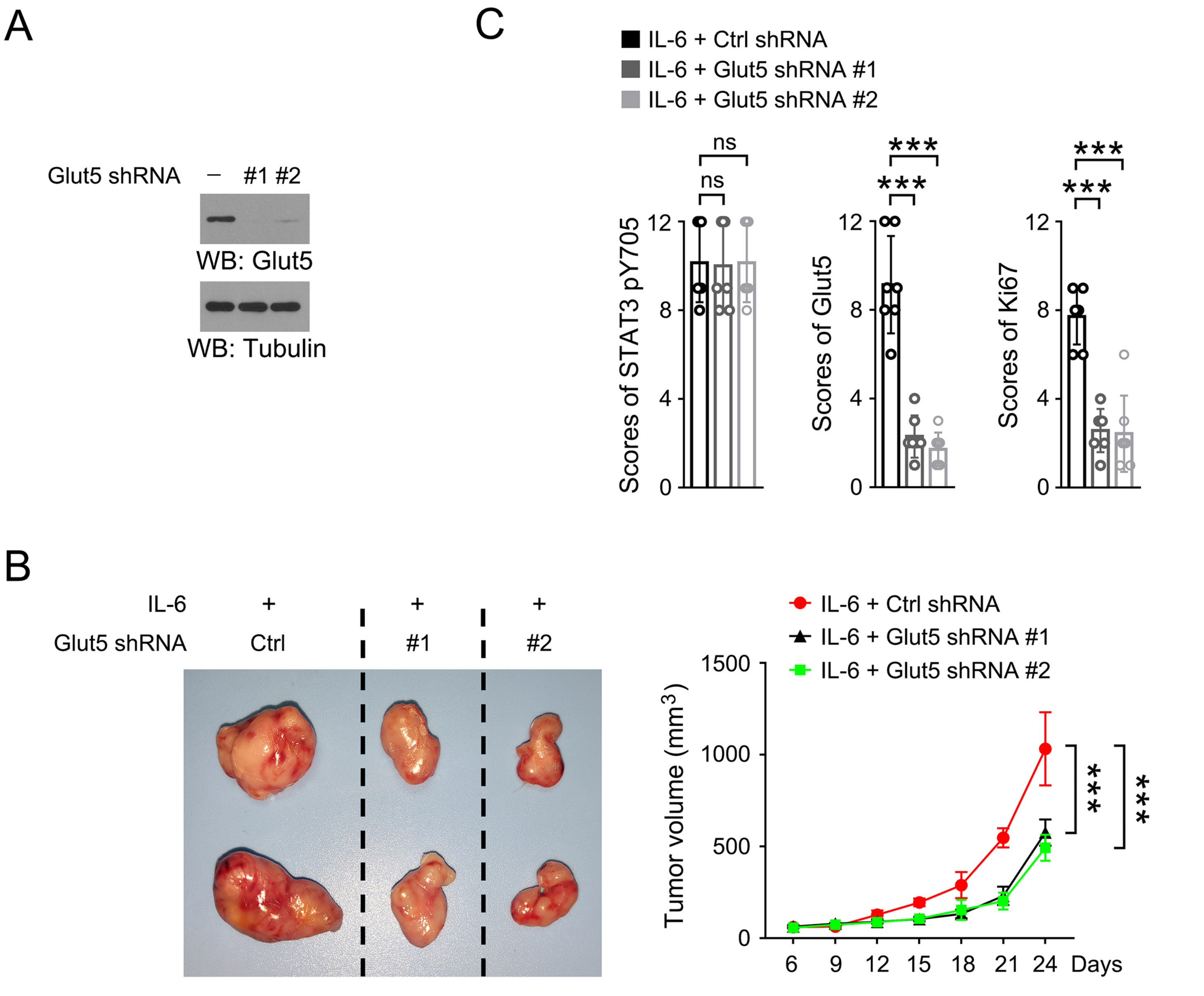

A
Glut5 shRNA
–
#1 #2
WB: Glut5
WB: Tubulin
C
IL-6 + Ctrl shRNA
IL-6 + Glut5 shRNA #1
IL-6 + Glut5 shRNA #2
ns
ns
***
***
***
***
Scores of STAT3 pY705
Scores of Glut5
Scores of Ki67
0
4
8
12
B
IL-6
+
+
+
Glut5 shRNA
Ctrl
#1
#2
IL-6 + Ctrl shRNA
IL-6 + Glut5 shRNA #1
IL-6 + Glut5 shRNA #2
Tumor volume (mm3)
0
500
1000
1500
6
9
12
15
18
21
24
Days
***
***

Figure S2

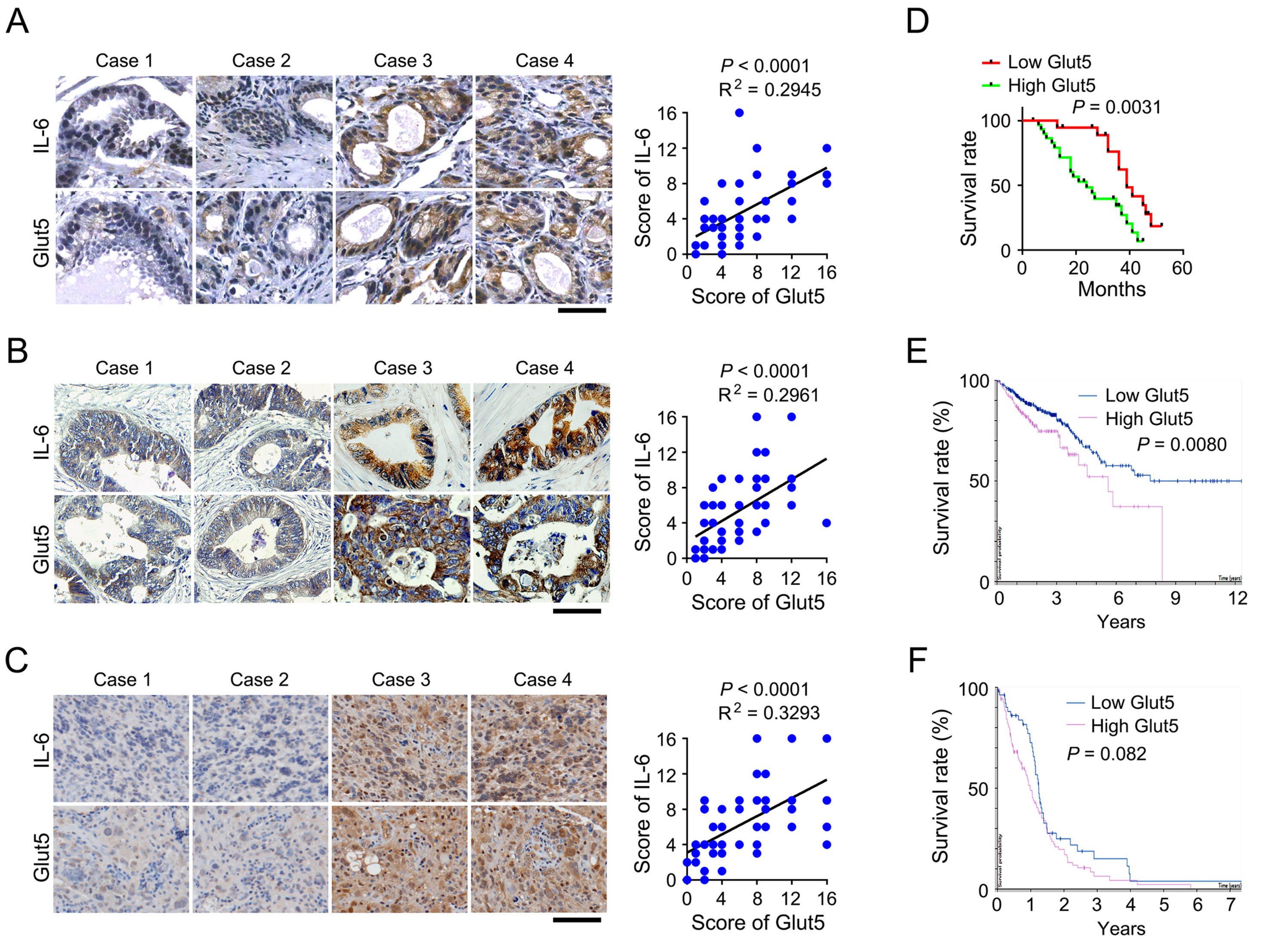

A
Case 1
Case 2
Case 3
Case 4
IL-6
Glut5
P < 0.0001
R2 = 0.2945
Score of IL-6
Score of Glut5
D
Low Glut5
High Glut5
P = 0.0031
Survival rate
Months
B
Case 1
Case 2
Case 3
Case 4
IL-6
Glut5
P < 0.0001
R2 = 0.2961
Score of IL-6
Score of Glut5
E
Low Glut5
High Glut5
P = 0.0080
Survival rate (%)
Years
C
Case 1
Case 2
Case 3
Case 4
IL-6
Glut5
P < 0.0001
R2 = 0.3293
Score of IL-6
Score of Glut5
F
Low Glut5
High Glut5
P = 0.082
Survival rate (%)
Years
